# Intelligent identification method of origin for Alismatis Rhizoma based on image and machine learning

**DOI:** 10.1038/s41598-025-98458-2

**Published:** 2025-04-23

**Authors:** Wenqi Zhao, Zongyi Zhao, Wen Zheng, Zimin Wang, Gaoting Yang, Zhiqiong Lan, Xiaoli Pan, Min Li

**Affiliations:** State Key Laboratory of Southwest Characteristic Chinese Medicine Resources, School of Pharmacy and College of Modern Chinese Medicine Industry, Chengdu University of Traditional Chinese Medicine, Chengdu, 611137 China

**Keywords:** Alismatis Rhizoma, Origins, Intelligent identification, Machine learning, Natural medicine, Medical research, Mathematics and computing

## Abstract

Alismatis Rhizoma (AR) is widely utilized as a natural medicine across many Asian countries. However, in China, due to its complex origins, AR quality varies, which can affect clinical efficacy. Therefore, there is a need for a method that is both fast and objective to determine the source of AR. In this study, a total of 400 samples of two species and four geographic origins from AR were imaged and processed. From these images, 17 features were extracted, including three shape (S), two color (C), and 12 texture features (T), resulting in a total of 6800 data points. Four commonly used classification models Random Forest (RF), Extreme Learning Machine (ELM), Back Propagation (BP) neural network, and Support Vector Machines (SVM) were tested to find the optimal combination of AR fusion features and classification models. The S + T-RF combinations achieved the best results, with 99.17% accuracy in two species identification and 96.67% accuracy in four geographic origin identification on test sets. These results suggest that image processing combined with the RF model can quickly and effectively identify the complex origins of AR and can provide a reference for the origins identification of other natural medicines.

**Supplementary Information:**

The online version contains supplementary material available at 10.1038/s41598-025-98458-2.

## Introduction

Alismatis Rhizoma (AR), also known as “Zexie” (泽泻) in Chinese, is a significant traditional medicinal herb widely used in China, Korea, Japan, and other East Asian countries^1,2^. For over 1800 years, AR has been commonly employed to treat conditions such as edema, hyperglycemia, hyperlipidemia, obesity, and nephritis^3,4^. Recent studies have also demonstrated that triterpenoid constituents in AR extracts exhibit anticancer activity against various cancer cell lines^5^. AR is derived from the dried tubers of two species in the Alismataceae family, and it has a long cultivation history in China as an important agricultural product, the primary producer, with annual production and sales volumes reaching up to 10,000 tons^6^. Cultivation is concentrated in four major regions: Sichuan, Fujian, Guangxi, and Jiangxi^7^. AR commodities are traditionally categorized into four types based on two plant species and four geographic origins: Chuan Zexie (cultivated in Sichuan Province) and Guang Zexie (cultivated in Guangxi Province) are both derived from *Alisma plantago-aquatica* Linn., while Jian Zexie (cultivated in Fujian Province) and Jiang Zexie (cultivated in Jiangxi Province) originate from *Alisma orientale* (Sam.) Juzep^7,8^. Modern research indicates that species differences and geographic variations in AR can lead to quality differences, which in turn impact clinical efficacy^9,10^. Consequently, effective, fast identification of AR species and geographic origin is essential for fair market transactions and reliable clinical applications.

Different varieties and origins of AR exhibit variations in physical characteristics. However, the current edition of the Pharmacopoeia of the People’s Republic of China lacks specific content in its quality standards for AR^2^. Consequently, in actual trade, identification relies entirely on the expertise of seasoned traditional Chinese medicine practitioners and professional appraisers, often leading to disputes. Modern research has revealed that, beyond surface traits such as the presence or absence of distinct transverse annular grooves, roughness, and tubercles, AR also displays subtle differences in shape and color^4,11,12^. Liu et al.^4^. found that “Chuan Zexie” is ovoid with a yellowish-brown surface, while “Jian Zexie” is subglobular or ellipsoidal with a yellowish-white surface. Yang et al.^11^. reported that “Chuan Zexie” is ellipsoidal and pale yellow, whereas “Jian Zexie” is ellipsoidal or elongated-ellipsoidal with a yellowish-white surface. Cai et al.^12^. conducted a detailed investigation of AR from four major origins, noting that Sichuan AR is subglobular with a grayish-yellow surface, Fujian and Jiangxi AR are ellipsoidal or subglobular with a yellowish-white surface, and Guangxi AR is ellipsoidal or ovoid with a yellowish-gray surface. However, these studies’ descriptions of physical characteristic variations across origins are inconsistent and remain qualitative, making them vague, difficult to standardize, and challenging to apply widely.

In recent years, an increasing number of studies have proposed analytical methods to distinguish AR species and geographic origins using metabolomics, genetics, chromatography, and spectroscopy. For instance, Zhang et al. utilized a combination of untargeted metabolomics, data cross-validation, absolute quantification, and vector machine modeling to identify AR’s geographic origins and plant species^13^. Lu et al. developed a pyrophosphate sequencing method to characterize different AR species through ITS2 sequence identification^14^. Yu et al. applied HPLC-DAD fingerprinting, multicomponent quantification, and pattern recognition to differentiate AR from various geographic origins^15^, while Zhao et al. employed near-infrared spectroscopy to distinguish AR’s primary species and origins in China^16^. Despite their accuracy, these advanced methods require sophisticated instruments and specialized personnel, making them costly, time-consuming, and challenging to apply in practical settings. Thus, developing an accurate and rapid method for identifying AR species and geographic origins is essential for promoting fair trade in the natural medicine market and ensuring safe clinical applications.

With advancements in modern image processing and machine learning algorithms, traditional manual identification methods based on morphological differences can now be digitized with high precision. This enables the creation of comprehensive image databases and the development of machine-trained classification models for accurate identification, driving progress in intelligent recognition technologies. These technologies offer high accuracy, rapid recognition, and the capacity to process large sample volumes at a low cost. As a result, their applications in natural medicines, food, and agriculture are expanding. For instance, Çetin et al.^17^. used image processing to extract size, shape, and area features of chickpea (*Cicer arietinum* L.) cultivars, combining these with machine learning algorithms to achieve accurate and rapid classification. Ropelewska et al.^18^. leveraged image color and texture features with machine learning to efficiently distinguish tomato seed cultivars. Koklu et al.^19^. applied machine vision to extract shape features of dried bean seeds, demonstrating that various machine learning algorithms could effectively differentiate dry bean varieties. These studies highlight the efficacy of combining image processing with machine learning for crop variety identification. Furthermore, integrating multiple features with machine learning can enhance classification performance. Cao et al.^20^. for example, used multispectral imaging to extract chroma, saturation, brightness, and texture features, building a classification model based on fused features that, combined with multiple machine learning methods, successfully classified tea plant varieties with precision. Yan et al.^21^. extracted HSI and texture features from drone imagery of pine trees affected by pine wood wilt disease, finding that combining two or more feature categories in feature space outperformed single-feature models across various machine learning approaches, significantly improving infected tree identification accuracy. Chen et al.^22^. developed a deep learning method to rapidly and accurately identify *fritillaria cirrhosa* species. In summary, both traditional machine learning and deep learning, when paired with image recognition, are effective for sample classification. Deep learning, such as deep learning, excels at automatic feature extraction and handling complex, high-dimensional data, making it ideal for large-scale image recognition and video analysis^23,24^. However, its high model complexity demands substantial data and computational resources, and its interpretability is limited. In contrast, traditional machine learning methods like Random Forest (RF) and Support Vector Machines (SVM) require fewer computational resources, offer greater interpretability, and are better suited for small datasets and simpler tasks. However, they struggle with high-dimensional, nonlinear data and exhibit weaker generalization^25,26^. He et al.^27^. for instance, extracted color space variations and texture features from small-sample wolfberries berry images using image processing and compared machine learning and deep learning approaches. Their results showed that RF outperformed deep learning in identifying wolfberries berry origins. Thus, traditional machine learning combined with image processing is a more efficient and cost-effective approach for identifying species and origins of traditional Chinese medicinal materials like AR, particularly with small datasets.

**Fig. 1:**
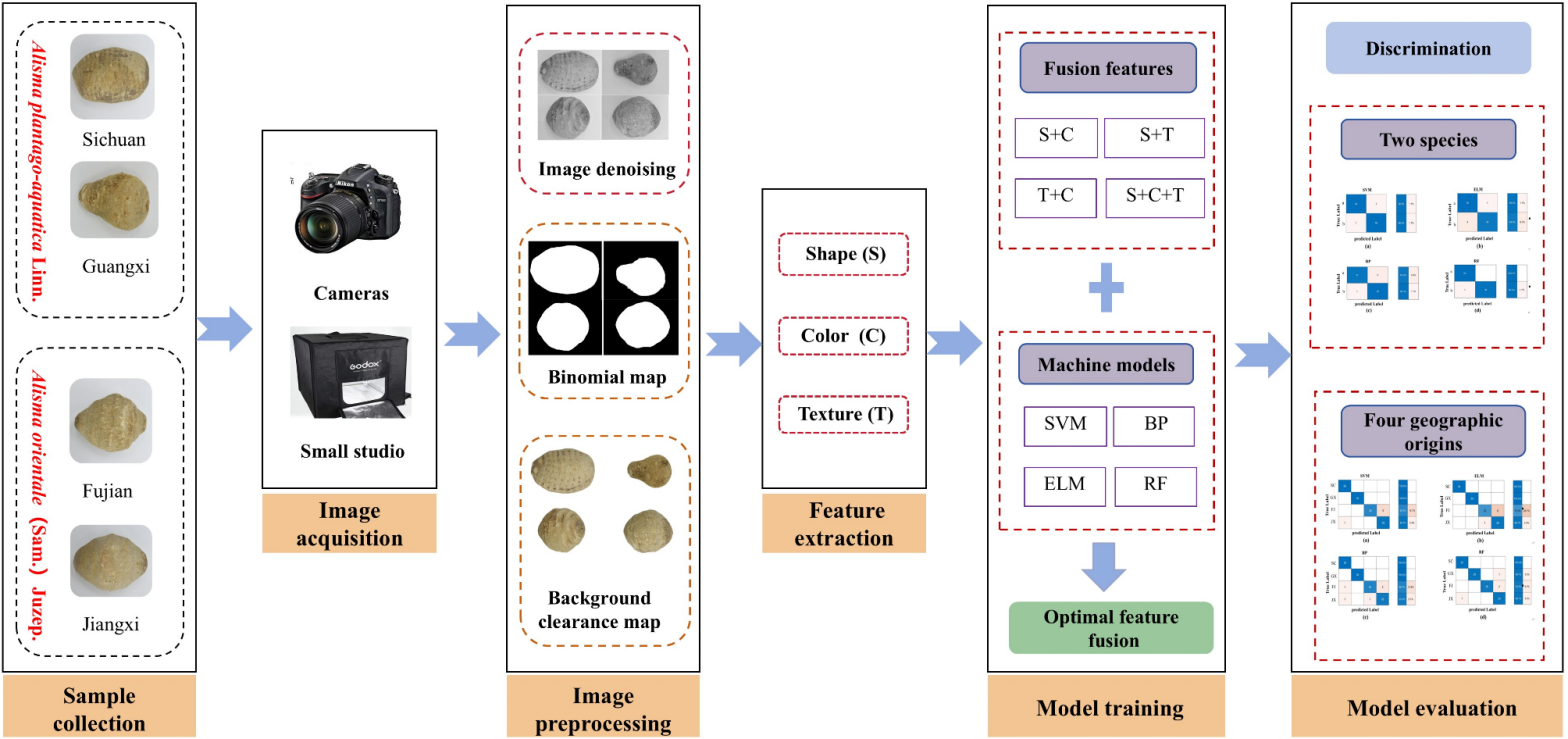
Flowchart of the proposed method.

The purpose of this study is to explore an intelligent method that combines modern image processing and machine learning techniques to identify the complex sources of AR. First, a large number of samples of AR from different species and geographical sources were collected. Secondly, high quality images of the AR samples were collected. Third, pre-process the images. Fourth, extract shape, color, and texture features of AR images. Fifth, combine the AR fusion features with the machine learning classification model to screen the best combination of AR fusion features and classification model. Finally, make a comprehensive performance evaluation of the best fusion features and classification model of AR. The specific process is shown in Fig. 1.

## Materials and methods

### Sample preparation and image acquisition

**Fig. 2:**
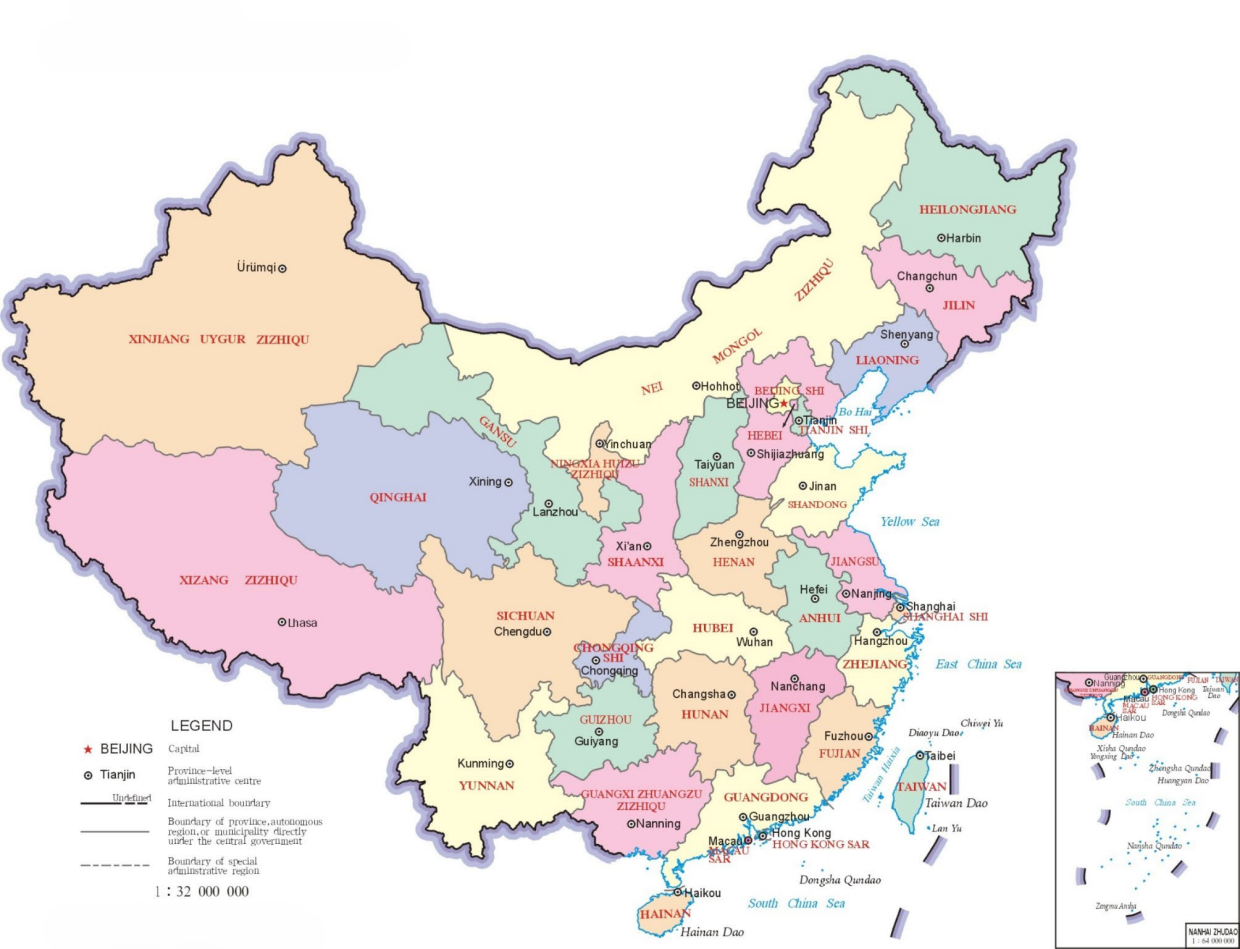
The four provinces shown, SICHUAN (Sichuan), GUANGXI ZHUANZUZIZHIQU (Guangxi), FUJIAN (Fujian), and JIANGXI (Jiangxi), are the geographic origin of Alismatis Rhizoma (AR); Sichuan and Guangxi correspond to *Alisma plantago-aquatica* Linn.; Fujian and Jiangxi correspond to *Alisma orientale* (Sam.) Juzep. The map of China in the image is sourced from the National Platform for Common Geospatial Information Service (https://bzdt.ch.mnr.gov.cn), with the map approval number GS (2019) 1680.

In this study, from 2022 to 2024, we collected 400 dried (AR) samples from the four major production regions in China: Sichuan (SC), Guangxi (GX), Fujian (FJ), and Jiangxi (JX). Notably, the AR species of AR from Sichuan and Guangxi is *Alisma plantago-aquatica* Linn., while the species of AR from Fujian and Jiangxi is *Alisma orientale* (Sam.) Juzep. Subsequently, 100 images were obtained from AR samples from each geographic origin, resulting in a total of 400 images (200 images from each species: *Alisma plantago-aquatica* Linn. and *Alisma orientale* (Sam.) Juzep.). All experimental samples were identified as the Chinese medicinal herb Alismatis Rhizoma by Professor of Pharmacy Yuecheng Li (Sichuan Institute for Drug Control, China) and associate Prof. Zhiqiong Lan (Chengdu University of Traditional Chinese Medicine, Chengdu, China). No fresh plant material was collected in this experiment. Voucher specimens of plant material were not made and all dry samples were purchased from cultivars in the main production areas in China. These dry experimental samples were stored in the Chinese Medicine Herbarium of Chengdu University of Traditional Chinese Medicine (CDCM). It should be noted that we complied with relevant institutional, national, and international guidelines and legislation for the experiment samples collected in this paper. The original information is presented in Table 1 and illustrated in Fig. 2.

To ensure consistency in image acquisition, photos were taken under uniform conditions using a professional adjustable LED light source shooting box (80 cm × 80 cm × 80 cm) with a shadowless LED lamp as the light source. A Nikon D7200 camera with an EF-S 18–200 mm f/3.5–5.6 IS STM lens was used. The camera settings were as follows: shutter speed of 1/80 s, aperture (F) of 8, focal length of 170 mm, and ISO sensitivity of 400. The camera was fixed directly above the sample, which was placed on a platform 30 cm below the lens. The image resolution was set to 6000 × 4000 pixels, and all images were saved in JPG format.

**Table 1:** Alismatis Rhizoma (AR) sample information sheet.

Species name	Geographical origin	The number of samples/pcs
*Alisma plantago-aquatica* Linn.	Meishan County, Sichuan Province	50
	Leshan County, Sichuan Province	50
	Guigang County, Guangxi Province	100
*Alisma orientale* (Sam.) Juzep.	Nanping County, Fujian Province	100
	Fuzhou County, Jiangxi Province	100

### Image preprocessing

**Fig. 3:**
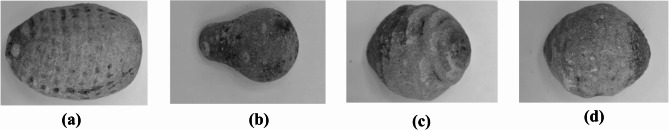
Background median filtered noise reduction maps for two species and four geographic origins of AR: **(a)** AR from Sichuan Province, China; **(b)** AR from Guangxi Province, China; **(c)** AR from Fujian Province, China; **(d)** AR from Jiangxi Province, China; **(a**, **b)** correspond to *Alisma plantago-aquatica* Linn.; **(c**, **d)** correspond to *Alisma orientale* (Sam.) Juzep.

**Fig. 4:**
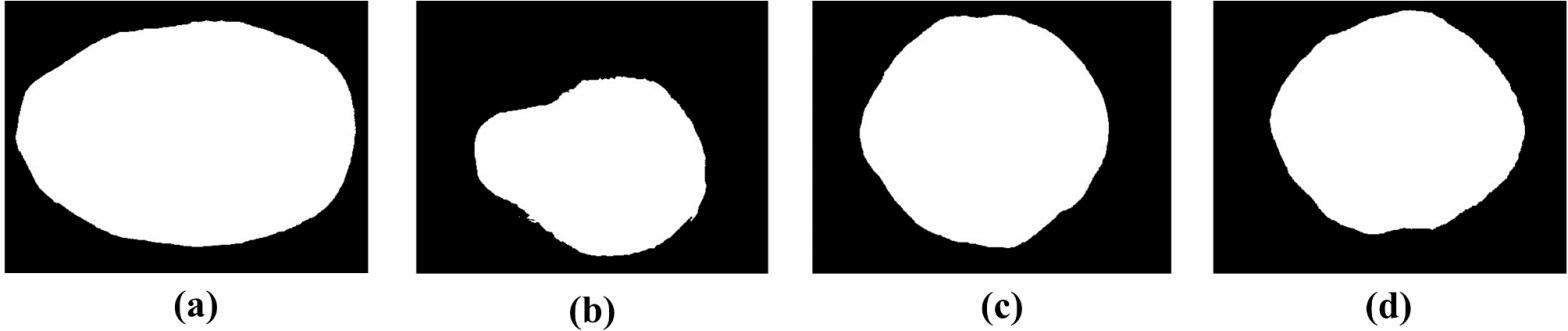
Binary map preprocessing for two species and four geographic origins of AR: **(a)** Chinese AR from Sichuan Province; **(b)** Chinese AR from Guangxi Province; **(c)** Chinese AR from Fujian Province; **(d)** Chinese AR from Jiangxi Province; **(a**, **b)** correspond to *Alisma plantago-aquatica* Linn.; **(c**, **d)** correspond to *Alisma orientale* (Sam.) Juzep.

**Fig. 5:**
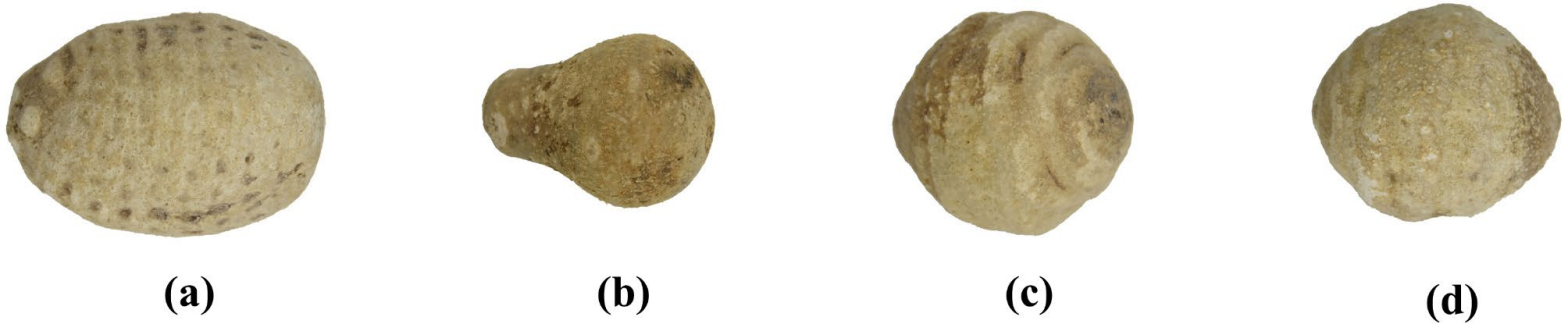
Background clearance map preprocessing of two species and four geographic origins of AR: **(a)** AR from Sichuan Province, China; **(b)** AR from Guangxi Province, China; **(c)** AR from Fujian Province, China; **(d)** AR from Jiangxi Province, China; **(a**, **b)** correspond to *Alisma plantago-aquatica* Linn.; **(c**, **d)** correspond to *Alisma orientale* (Sam.) Juzep.

During the acquisition and transmission process, AR images may be affected by various sources of environmental noise and electronic interference, resulting in a complex background. Therefore, it is necessary to preprocess the AR images to enhance image clarity, accuracy, and reliability, thus ensuring optimal conditions for subsequent feature extraction. First, median filtering is applied to denoise the images (Fig. 3). Next, the RGB image is converted to the HSV color space, and the S-channel is segmented into a binary image based on the saturation difference between the background and the target, with voids in the target area filled (Fig. 4). Finally, the pixel values of the R, G, and B channels are set to 255, and the three channels are merged to produce an image with a pure white background for subsequent AR feature extraction (Fig. 5).

### Extraction features

In this study, shape, color, and texture features were extracted from 400 AR images, and the resulting feature values were stored in an Excel file for subsequent analysis.

#### Extraction of shape features

The binary image targets of 400 AR images were labeled and measured using the bwlabel and regionprops functions in the digital image processing toolbox of MATLAB software. Five parameters were obtained: Area, Perimeter, MajorAxisLength, MinorAxisLength, and BoundingBox. Subsequently, inspired by the Eqs. (1), (2) and  (3), the aspect ratio, rectangularity, and circularity values of the AR images were calculated as follows^28^:

1 $${\text{C}} = {\text{4}}\pi {\text{A/P}}^{{\text{2}}}$$

2 $${\text{R}} = {\text{A}}/{\text{A}}_{{\text{R}}}$$

3 $${\text{L }} = {\text{ J}}/{\text{I }}$$

where A = area; P = perimeter; A_R_ = area of the minimum outer join matrix; J = long axis and I = short axis.

#### Extraction of color features

**Fig. 6:**
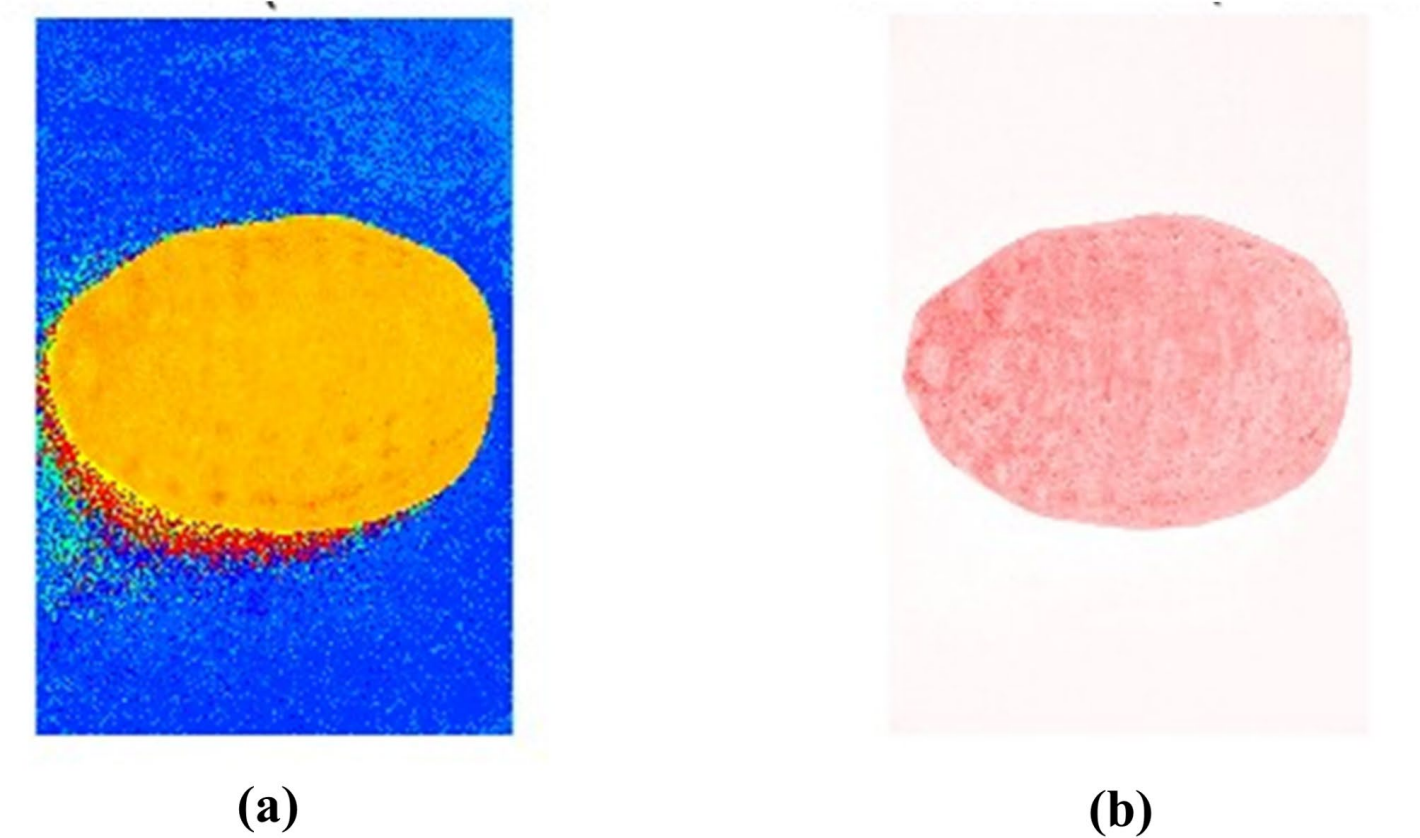
H and S two-channel information on the AR (Sichuan); **(a)** H-channel image; **(b)** S-channel image.

Currently, RGB and HSV are commonly used color spaces in image processing. Compared to RGB, HSV can more intuitively represent specific color information such as hue (H), saturation (S), and luminance (V). The H and S components align more closely with human perception of color, whereas the V component is more sensitive to changes in illumination. In many applications, variations in luminance have a smaller impact on color recognition and segmentation than the H and S components. Therefore, in this study, 400 AR images with backgrounds removed in Step “2.2”. were converted to the HSV color space. The mean2 function was then applied to extract the mean values of the H and S channels as the color features of AR (Fig. 6).

#### Extraction of texture features

Texture feature extraction is a critical aspect of image analysis, employing image processing techniques to compute eigenvalues that provide quantitative or qualitative descriptions of texture. Texture features based on the gray-level co-occurrence matrix (GLCM) were proposed by Haralick and Dinstein as a mathematical model^29^. This model calculates the frequency of occurrence of a gray-level pair P (x, y∣d,θ) formed by a pixel with gray-level value i and another pixel with gray-level value j at a distance (dx, dy), represented as a square matrix P (x, y∣d,θ). This matrix, called the grayscale co-occurrence matrix, has dimensions G × G, where G denotes the number of gray levels in the image. The parameters dx and dy are defined by the distance *d* and angle *θ* between the two pixels. The mathematical expression is Eq. (4)^30^.

The gray-level co-occurrence matrix (GLCM) provides information about an image’s orientation, spacing, range of variation, and texture frequency; however, it does not directly characterize different textures. Therefore, it is necessary to extract statistical values to quantitatively describe texture features based on the GLCM. In this study, commonly used texture feature statistics for this purpose include Angular Second Moment (ASM) in Eq. (5), Contrast (CON) ) in Eq. (6), Correlation (COR) in Eq. (7), and Entropy (ENT) in Eq. (8)^31,32^.

4 $${\text{P}}({\text{i}},{\text{j|d}},\theta ){\text{ = }}\{ ({\text{x}},{\text{y}}){\text{|f}}({\text{x}},{\text{y}}){\text{ = i}},{\text{f}}({\text{x + dx}},{\text{y + dy}}){\text{ = j}}; \ldots {\text{x}},{\text{y}} = 0,{\text{1}},{\text{2}},{\text{3}},....{\text{N}} - {\text{1}}\}$$

5 $$ASM = \:\sum\nolimits_{j} {\sum\nolimits_{i} {P(i,j)^{2} } }$$

6 $$CON = \:\sum\nolimits_{j} {\sum\nolimits_{i} {(i - j)^{2} P(i,j)} }$$

7 $${\text{COR}} = |\sum\nolimits_{j} {\sum\nolimits_{i} {\left( {{\text{i}},{\text{j}}} \right){\text{P}}\left( {{\text{i}},{\text{j}}} \right) - \mu _{{\text{x}}} \mu _{{\text{y}}} } } |{\text{ }}/\partial _{{\text{x}}} \partial _{{\text{y}}}$$

8 $${\text{ENT}} = \sum\nolimits_{j} {\sum\nolimits_{i} {{\text{P}}\left( {{\text{i}},{\text{j}}} \right)*\left( {{\text{log}}\left( {{\text{P}}\left( {{\text{i}},{\text{j}}} \right)} \right)} \right)} }$$

### Screening of the features and the establishment of the identification models

In this study, 400 AR images were divided into training and test sets at a 7:3 ratio, with 210 samples randomly selected for the training set and the remaining 90 samples for the test set. The four classification models (BP, SVM, RF, and ELM)—were combined with shape, color, and texture fusion features to identify the optimal combination.

### Models evaluation

Confusion matrices provide a visual method of model evaluation, accurately and intuitively presenting the prediction results for each category. In this study, to further assess the ability of the best model to recognize each category, a confusion matrix was used for in-depth evaluation. The tests were conducted using k-fold cross-validation with k = 10.

To minimize the risk of over fitting and to evaluate the models more reasonably and accurately, accuracy in Eq. (9), precision in Eq. (10), recall in Eq. (11), and F1-score in Eq. (12) were used as evaluation metrics in this study^33,34^. These indicators reflect different aspects of model classification performance, with higher values indicating better model performance. The calculation formulas are provided below. The F1-score metric combines the outputs of precision and recall.

9 $${\text{Accuracy}} = \left( {{\text{TP}} + {\text{TN}}} \right)/\left( {{\text{TP}} + {\text{FP}} + {\text{TN}} + {\text{FN}}} \right) \times {\text{1}}00\%$$

10 $${\text{Precision = TP / (TP + FP) }} \times {\text{100\% }}$$

11 $${\text{Recall = TP / (TP + FN) }} \times {\text{100\% }}$$

12 $${\text{F1}} - {\text{score}} = {\text{2Precision}} \times {\text{Recall}}/\left( {{\text{Precision }} + {\text{ Recall}}} \right) \times {\text{1}}00\%$$

where TP = true positives; TN = true negatives; FP = false positives and FN = false negatives.

In this experiment, the aforementioned algorithms were implemented using MATLAB 2021a (MathWorks Inc., Natick, MA, USA). The system specifications include an Intel Core i9-13900 H CPU, 64 GB of DDR5 5600 MHz RAM, with a base frequency of 5.2 GHz, running on the Windows 11 operating system.

## Results

### Selection of the best fusion features and classification models for AR

In this study, 400 images of AR samples were subjected to feature extraction, with 17 features extracted from each image. These features included three shape, two color, and 12 texture features, resulting in a total of 6,800 data points. See attached drawing (1–3).

#### Combination of fusion characteristics and taxonomic models for two species of AR

The classification performance of single features for two AR species was evaluated using RF, SVM, BP, and ELM models. Results (Table 2) indicate that feature S, when used alone, achieved test accuracies of 83.33–87.50% across the four models, demonstrating good performance. Feature C yielded lower accuracies (70.83–80.00%), suggesting a limited contribution to classification. Conversely, feature T consistently achieved accuracies above 95.83%, indicating it is a strong discriminator for this task.

In multi-feature fusion experiments, different AR feature combinations influenced the test accuracies of BP, RF, SVM, and ELM models. The S + T combination yielded accuracies of 97.50–99.17%, reflecting high classification performance. The S + C combination achieved 89.17–96.17%, showing moderate improvement over single features but underperforming compared to S + T. The C + T combination resulted in accuracies of 95.00–96.67%, indicating solid performance in certain scenarios. The S + C + T combination, encompassing all features, produced accuracies of 93.33–97.50%. Despite including all feature types, its performance did not significantly surpass partial combinations, possibly due to feature redundancy or interference.

Overall, multi-feature fusion outperforms single-feature approaches in classification performance. Among the four models, RF with the S + T combination achieved the highest accuracy in both training and test sets. Thus, this study selects the S + T-RF combination as the optimal model for classifying two AR species.

**Table 2:** Comparison of taxonomic models for different fusion traits of two species of AR.

Models	Fusion feature	Feature number/pcs	Training set accuracy/%	Test set accuracy/%
BP	S	3	91.79	85.83
	C	2	84.29	80.00
	T	12	99.64	100.00
	S + T	15	99.64	97.50
	S + C	5	97.86	96.67
	C + T	14	99.29	96.67
	S + C + T	17	99.67	96.67
RF	S	3	93.57	83.33
	C	2	74.29	74.29
	T	12	100.00	96.67
	S + T	15	100.00	99.17
	S + C	5	99.17	95.00
	C + T	14	100.00	97.50
	S + C + T	17	100.00	96.67
SVM	S	3	85.36	86.67
	C	2	73.93	70.83
	T	12	100.00	95.83
	S + T	15	98.21	96.67
	S + C	5	97.50	95.00
	C + T	14	95.00	95.00
	S + C + T	17	99.29	98.33
ELM	S	3	89.29	87.50
	C	2	82.50	80.00
	T	12	96.79	96.67
	S + T	15	96.67	93.17
	S + C	5	92.86	89.17
	C + T	14	97.50	96.67
	S + C + T	17	94.64	93.33

S + T, means Shape and Texture features; S + C, means Shape and Color features; T + C; means Texture and Color features; S + C + T, means Shape, Color and Texture features.

#### Combination of fusion characteristics and taxonomic models for four geographic origins of AR

In single-feature experiments (Table 3), feature S showed relatively low accuracies across BP (79.17%), RF (75.83%), SVM (78.33%), and ELM (78.33%), suggesting a minor contribution to classification. Feature C (color) consistently yielded the lowest accuracy (73.21%) in both training and test sets, reinforcing its limited utility alone. Feature T, however, exhibited high test accuracies (above 95.00%) across all models, underscoring its critical role in geographical classification.

In multi-feature fusion experiments, the S + C combination produced test accuracies of 86.67–91.67%, the C + T combination 86.67–94.17%, and the S + C + T combination 91.67–95.00%. The S + T combination achieved 90.83–96.67%, demonstrating robust performance. These findings highlight the significant impact of feature combination choices on model performance, with varying advantages across models.

In conclusion, multi-feature fusion significantly outperforms single-feature methods for classifying four AR geographical origins. Among the four models, RF with the S + T combination achieved the highest accuracy in both training and test sets. Thus, this study adopts the S + T-RF combination as the optimal model for identifying four AR origins.

**Table 3:** Comparison of taxonomic models for different fusion characteristics of four geographic origins of AR.

Models	Fusion feature	Feature number/pcs	Training set accuracy/%	Test set accuracy/%
BP	S	3	80.00	79.17
	C	2	76.79	74.17
	T	12	97.86	95.00
	S + T	15	97.50	95.00
	S + C	5	91.43	91.67
	C + T	14	98.57	94.17
	S + C + T	17	98.57	93.33
RF	S	3	100.00	75.83
	C	2	99.29	70.00
	T	12	100.00	95.00
	S + T	15	100.00	96.67
	S + C	5	100.00	90.83
	C + T	14	100.00	94.17
	S + C + T	17	100.00	92.50
SVM	S	3	82.86	75.83
	C	2	73.21	68.33
	T	12	95.00	91.67
	S + T	15	98.93	94.17
	S + C	5	93.57	89.17
	C + T	14	96.07	92.50
	S + C + T	17	95.71	95.00
ELM	S	3	82.50	78.33
	C	2	75.36	81.67
	T	12	96.07	95.00
	S + T	15	92.14	90.83
	S + C	5	88.57	86.67
	C + T	14	93.21	86.67
	S + C + T	17	95.00	91.67

#### Statistical analysis

In this study, one-way analysis of variance (ANOVA) was employed to assess the inter-group differences in classification accuracy among four distinct models based on the combined features of S and T. The ANOVA results indicated no statistically significant differences (*p* > 0.05). Furthermore, 95% confidence intervals (CIs) were calculated for each model, as exemplified by the S + T-RF model: 95% CI [0.92, 0.96]. In summary, these findings suggest that there are no significant statistical differences in accuracy across the models. However, this does not imply that the models are equivalent in performance. Further analysis using larger datasets and more comprehensive performance metrics may be required to elucidate potential differences.

### Evaluation of identification models for AR species and geographic origins

#### Confusion matrix evaluation of different traditional classification models

**Fig. 7:**
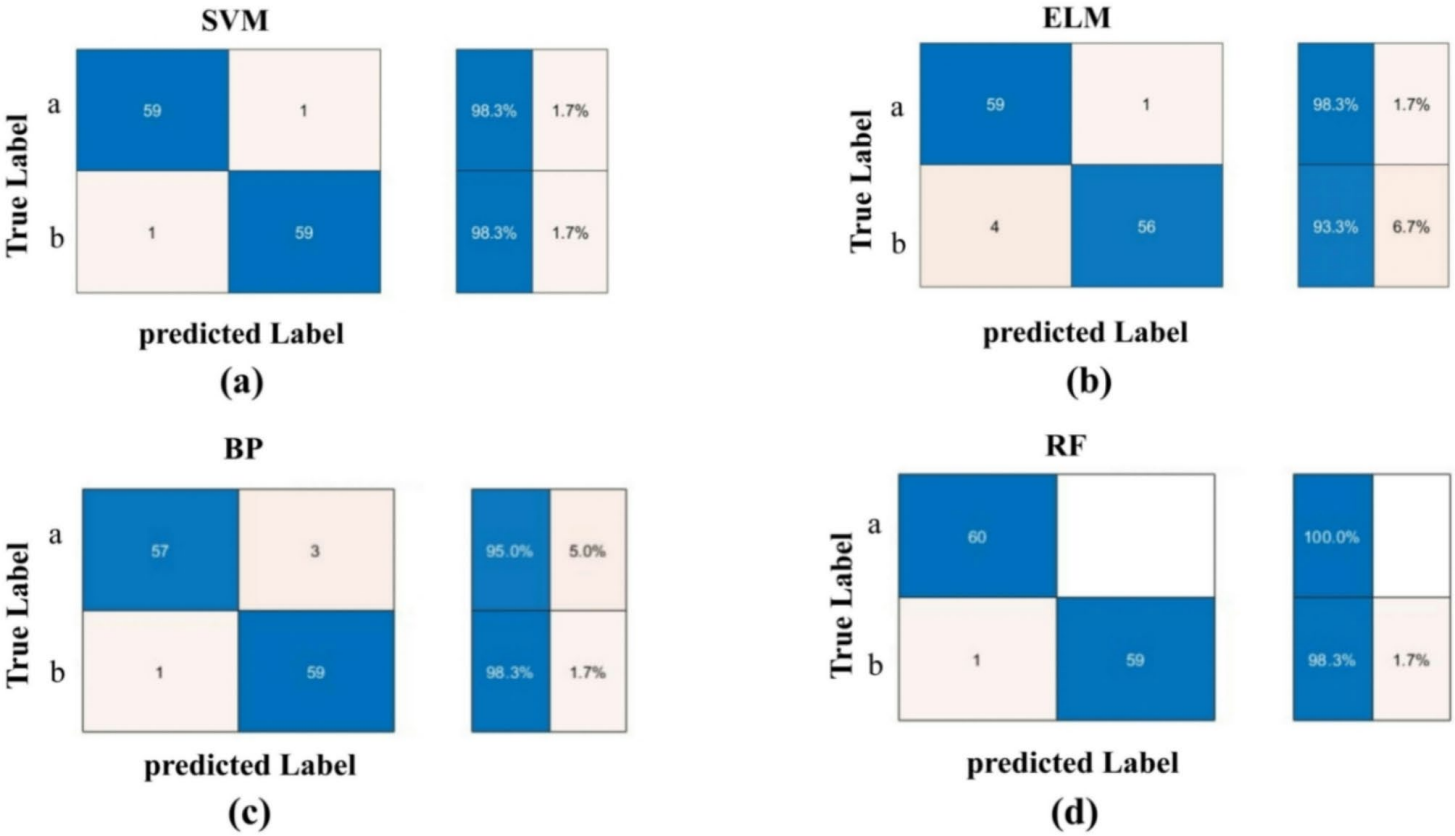
AR two species test set confusion matrices for **(a)** SVM; **(b)** ELM; **(c)** BP; and **(d)** RF.

**Fig. 8:**
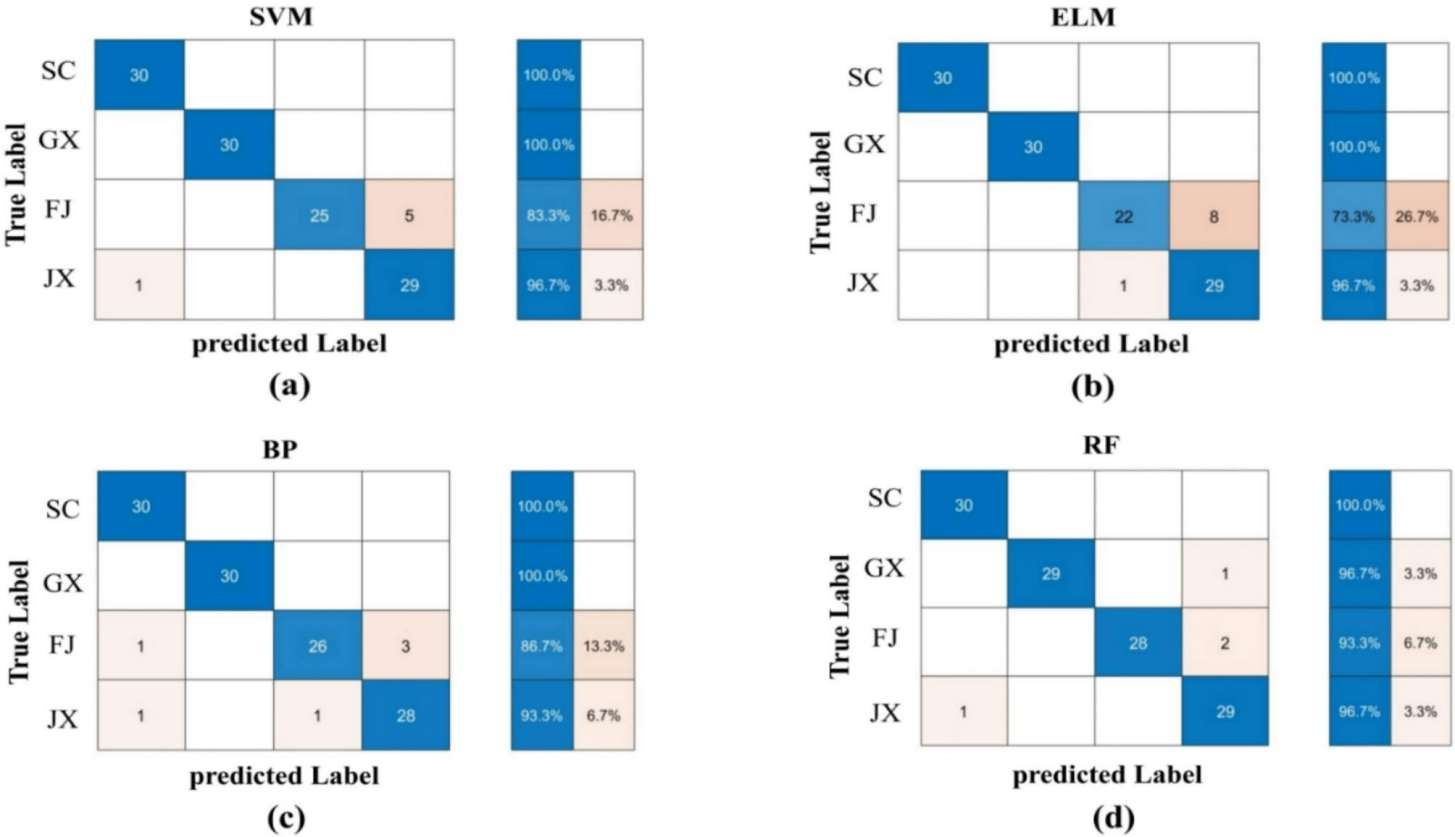
AR four geographic origins test set confusion matrices for **(a)** SVM; **(b)** ELM; **(c)** BP; and **(d)** RF.

In this study, through 10-fold cross-validation, we assessed the generalization capability and stability of the classification models. The results demonstrated that the S + T-RF combination achieved an average accuracy of 95% across the 10 validation runs. Confusion matrices were used to evaluate model performance for distinguishing two AR species and four origins. For the two species (Fig. 7), SVM, BP, and ELM achieved recognition rates of 98.30%, 96.65%, and 95.80%, respectively, while RF exceeded 99.15%, indicating superior discriminative ability. For the four origins (Fig. 8), BP recognized Fujian and Jiangxi AR at 86.70% and 96.30%, respectively, with 100% for other regions; RF achieved 100% for Sichuan and over 90% elsewhere; SVM scored below 90% for Fujian, above 96% for Jiangxi, and 100% elsewhere; ELM reached 100% for Sichuan and Guangxi, 83.30% for Fujian, and 96.70% for Jiangxi. Overall, RF outperformed SVM, ELM, and BP in recognizing both species and origins.

Accuracy, precision, recall, and F1-score were used to assess model performance (Table 4). For two AR species, all four models exceeded 93.33% across metrics, with RF achieving 100%, demonstrating optimal discriminative ability. For four origins (Table 5), RF outperformed others with 96.67% accuracy, 96.85% precision, 96.67% recall, and 96.69% F1-score. SVM and BP performed well but fell short of RF, while ELM scored lowest across metrics.

In summary, the four models established in this study, SVM, RF, ELM and BP, were all able to effectively recognize the two species and four geographic origins of AR, with the RF classification model performing most prominently.

#### Assessment of classification indicators for different traditional classification models

In this study, we evaluated the performance of classification models for discriminating between two species and four geographical origins of AR, using accuracy, precision, recall, and F1-score as metrics. The results showed that the S + T-RF model achieved an average accuracy of 96.67%, indicating the model’s high stability.

As shown in Table 4, when discriminating between the two species of AR, the accuracy, precision, recall, and F1 score of the SVM, BP, ELM, and RF models were all above 93.33%. Among them, the RF model achieved 100% in accuracy, precision, recall, and F1-score, demonstrating the best performance in discriminating the two species of AR.

As can be seen from Table 5, in identifying the four geographic origins of AR, compared to SVM, ELM, BP, and RF classification models, the RF classification model is the best in accuracy, precision, recall and F1-Score with 96.67%, 96.85%, 96.67%, 96.69%, respectively. SVM and BP perform well in their 4 evaluation metrics but are lower than RF classification model. ELM model has the lowest accuracy in this accuracy, precision, recall and F1-Score metrics.

**Table 4:** Results of the test set of taxonomic evaluation for the identification of two species of AR.

Models	Test set (%)				
	Class	Accuracy (%)	Precision (%)	Recall (%)	F1-score (%)
BP	a	96.67	98.28	95.00	96.61
	b		95.16	98.33	96.72
RF	a	99.17	98.36	100.00	99.17
	b		100.00	98.33	99.16
SVM	a	98.33	98.33	98.33	98.33
	b		98.33	98.33	98.33
ELM	a	95.83	93.65	98.33	95.93
	b		98.25	93.33	95.73

**Table 5:** Results of the test set of taxonomic evaluation for identification of four geographic origins of AR.

Models	Test set (%)				
	Class	Accuracy (%)	Precision (%)	Recall (%)	F1-score (%)
BP	SC	95.00	93.75	100.00	96.77
	GX		100.00	100.00	100.00
	FJ		96.30	86.67	91.23
	JX		90.32	93.33	91.80
RF	SC	96.67	96.77	100.00	98.36
	GX		100.00	96.67	98.31
	FJ		100.00	93.33	96.55
	JX		90.62	96.67	93.55
SVM	SC	95.00	96.77	100.00	98.36
	GX		100.00	100.00	100.00
	FJ		100.00	83.33	90.91
	JX		85.29	96.67	90.62
ELM	SC	92.50	100.00	100.00	100.00
	GX		100.00	100.00	100.00
	FJ		95.65	73.33	83.02
	JX		78.38	96.67	86.57

#### Deep learning vs. traditional classification methods

**Fig. 9:**
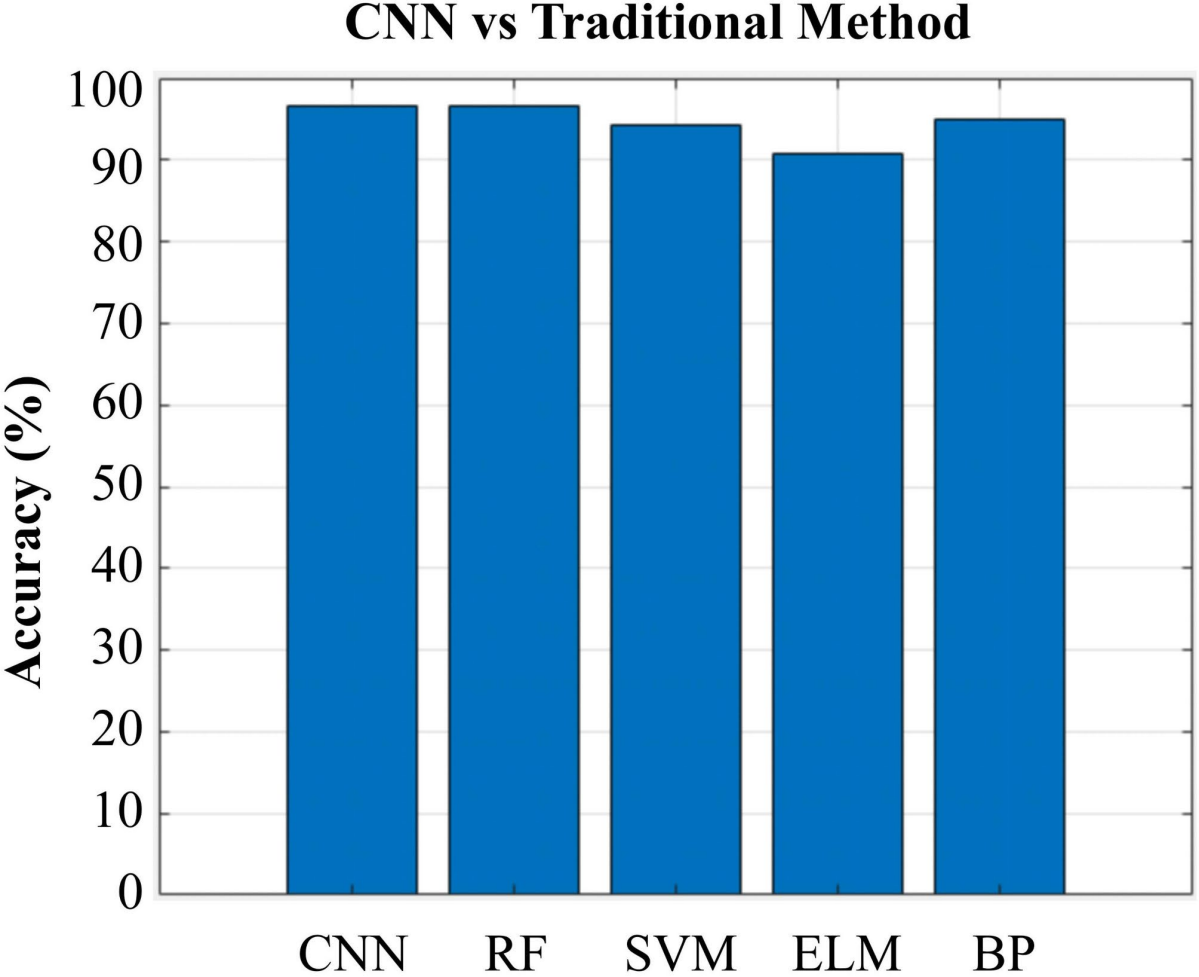
Comparison of test set accuracy between CNN and four traditional methods for AR with four geographical origins.

**Fig. 10:**
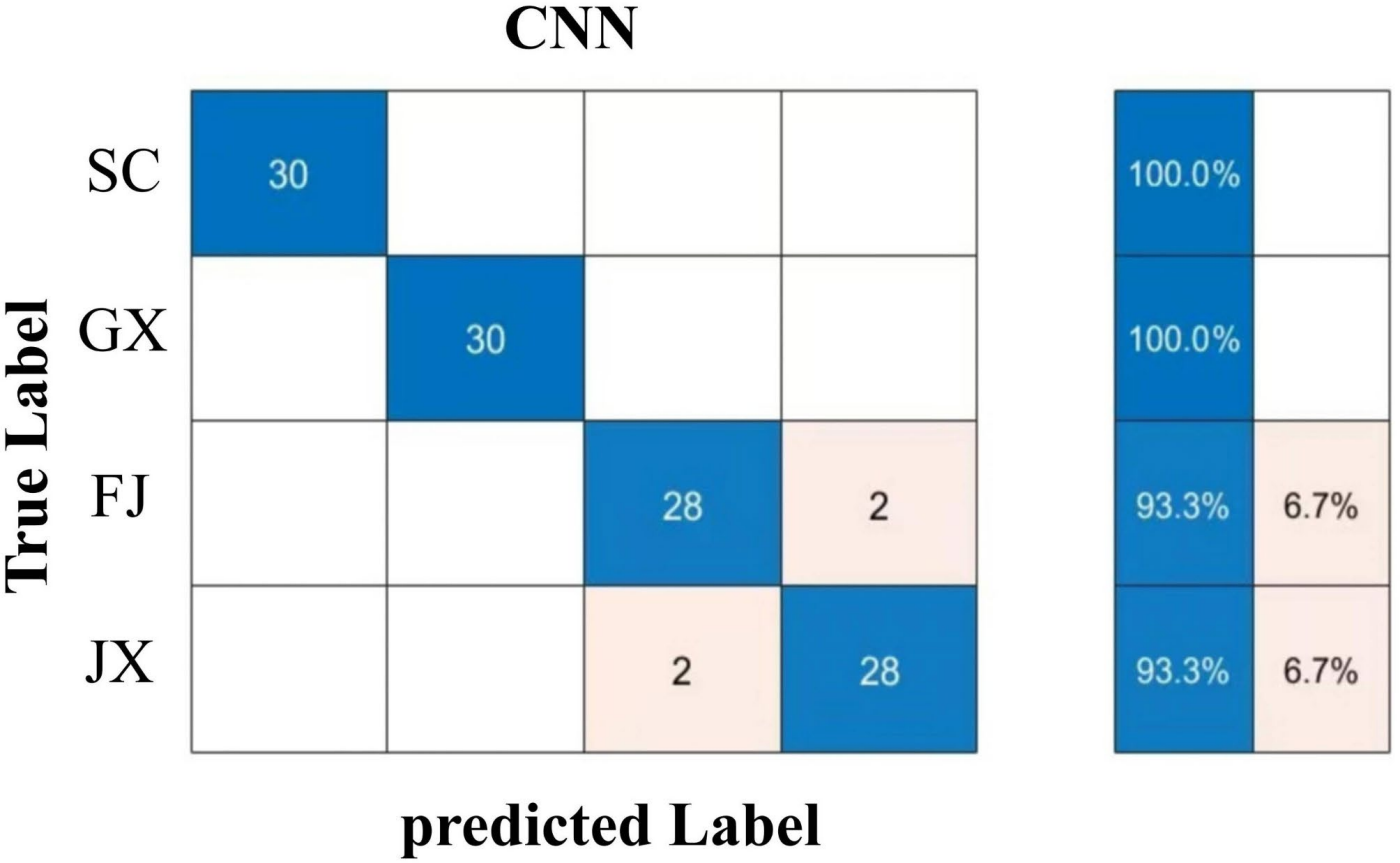
Confusion matrix of CNN test set for AR from four geographical origins.

To evaluate the performance differences among various classification methods, a comparison was made between the deep learning CNN and traditional machine learning methods such as BP, RF, SVM, and ELM. The AlexNet pre-trained model in CNN was employed, and data augmentation operations including rotation (± 30°) and flipping were performed. In light of the aforementioned results indicating that S + T-RF excels in the classification and identification of the two species of AR, this study solely compares the CNN model with traditional machine learning methods for the four geographical origins of AR. From the performance comparison bar chart (Fig. 9), in the classification task of AR origins, both CNN and RF achieved accuracy rates above 95%. Further analysis of the CNN test set confusion matrix, taking the four geographical origins of AR as an example, shows that the recognition rates for AR from Sichuan and Guangxi were 100%, while those from Fujian and Jiangxi were both 93.33%. This indicates that CNN classification capability varies across different categories (Fig. 10).

## Discussion

The species and geographical origin of natural medicines are important factors influencing their external properties, intrinsic quality, and clinical efficacy^9,10^. The appearance characteristics of AR from different sources vary, so image processing techniques can be applied and accurate and robust classification models can be developed to recognize AR herbs from different species and geographical origins. Currently, the main classification models commonly used in similar identification studies are RF, BP, SVM and ELM. RF is an integrated learning method that makes classification or regression predictions by constructing multiple decision trees and selecting the class with the highest number of votes as the final prediction for a new sample. However, RF may suffer from sample imbalance, and the model is not as explanatory as a single decision tree^35^. ELM is a single-layer forward neural network based on randomization, where the training process does not require iterative corrections. This reduces the number of computations and provides fast learning speed, high accuracy, and easy implementation^36^. SVM finds optimal hyperplanes in high-dimensional spaces by mapping the input space to a high-dimensional feature space using kernel functions, and are suitable for small and high-dimensional data, especially linearly indivisible data^37^. The BP neural network is a multilayer feedforward network trained with an error back propagation algorithm. As an algorithm with highly nonlinear mapping ability, it has better classification and recognition capabilities for complex patterns. However, it is prone to issues like local minima and overfitting^38^. Therefore, each model has its specific advantages and limitations, which need to be selected and adjusted according to the research needs and the characteristics of the specific research object in the practical application.

The results of this study on identifying two species and four geographic origins of AR based on the combination of the best appearance fusion features S + T show that RF is the most effective in identifying both two species and four geographic origins of AR compared to the other three classification models BP, SVM, and ELM, and all of its evaluation indexes are greater than 90.00%. Modern studies have also found that RF can be widely used in image and spectral feature extraction for classification and identification studies of agricultural products and natural herbs. For example, He et al.^27^. realized intelligent identification of Wolfberries origin by extracting color space transformation and texture morphology features combined with RF based on image processing. Notably, when identifying the two species of AR, the test accuracy of the S + T combination based on fused features was higher than that of the S + T + C full-feature combination in all four classification models, namely, RF, BP, SVM, and ELM. When identifying its four geographic origins, the S + T fusion features also had higher test accuracies than the S + T + C full-feature combination in both BP and RF classification models. This suggests that increasing the number of features does not necessarily improve performance; on the contrary, it may introduce redundancy, which can reduce classification accuracy, as demonstrated in several studies. Yan et al.^21^. compared a single feature or a combination of two or two features of pine wilt disease infected trees, and the classification accuracy obtained using all feature combinations was not the highest in the RF, CatBoost and KNN models. Khojastehnazhand et al.^39^. improved the correct classification rate of the test dataset to 98.10% by selecting 20 features through the chi-square test feature selection method, which outperformed the modeling results of all extracted features. Therefore, reasonable selection and fusion of features are needed to optimize the model performance in practical applications.

This study focuses on feature extraction and classification of small-sample AR species and origins, opting for traditional features (shape, color, texture) over sole reliance on CNN for several reasons. First, traditional features offer strong interpretability, directly correlating with AR’s biological traits—e.g., texture reflects epidermal microstructures, aiding botanical classification. Second, the limited dataset (400 images) restricts CNN training efficacy, while traditional methods perform reliably on small data. Third, computational efficiency is critical; MATLAB’s toolchain excels in processing small-to-medium datasets, enabling rapid iteration and enhancing research efficiency. Experiments comparing traditional machine learning (SVM, RF, BP, ELM) with CNN showed comparable accuracy, validating the feasibility of traditional methods for small datasets with limited computational resources. Literature suggests small datasets often limit model generalization, risking overfitting and reducing performance on unseen data^25^. Here, carefully designed feature extraction and ablation experiments maximized representative feature extraction, boosting generalization. CNN, however, are prone to overfitting on small datasets, diminishing generalization. Given that traditional Chinese medicine identification often involves small samples and resource-constrained settings (e.g., herbal markets), traditional machine learning offers advantages in rapid deployment and validation. For large-sample, complex-origin materials like chrysanthemum and Dendrobium, we plan to explore a “traditional features + lightweight CNN” hybrid model, leveraging traditional feature interpretability and efficiency alongside lightweight CNN feature learning to address complex source identification.

## Conclusions

Compared to other types of medicines, natural medicines are characterized by a wide variety of species, complex origins, and significant differences in quality^40,41^. Both traditional experience and modern research have shown that the appearance characteristics of natural medicines are closely related to their species and geographic origins^42^. However, most identification methods remain limited to intuitive human assessments, relying on sensory perceptions such as sight, smell, and taste. With the rapid development of image technology and machine learning, it is now possible to extract morphological features, such as shape and texture, from natural medicines to enable intelligent recognition of their species and geographic origins^43^.

AR is a natural medicine commonly used in Chinese medicine, and its species and geographic origin significantly influence its medicinal value. In this study, shape, color, and texture features of AR samples from two species and four different geographic origins were extracted using image processing techniques. The optimal classification model and feature fusion combination for AR were identified by comparing the performance of four models—SVM, RF, ELM, and BP—combined with the fusion features of AR. The results demonstrated that the S + T-RF combination achieved the highest accuracy, precision, recall, and F1-score in identifying the two species and four geographic origins of AR, outperforming other fused feature and model combinations. This method enables objective, rapid, and efficient identification of AR’s species and geographic origin. This study provides a valuable reference for advancing artificial intelligence recognition in the identification of other natural medicines with complex sources. It also promotes the scientific application of modern computer and electronic technologies in the quick inspection method study of traditional natural medicines. Future work should focus on continuously updating and expanding the AR sample set to include additional geographic origins, such as emerging production areas, thereby creating a larger AR image recognition database. Further efforts should also be directed toward developing applications with faster recognition speeds and simpler, more user-friendly interfaces to facilitate convenient identification of AR commodities.

## Electronic supplementary material

Below is the link to the electronic supplementary material.

Supplementary Material 1.

## Data Availability

Data is contained within the article or supplementary material.
